# A self‐insertion of the styrofoam in urinary bladder and literature review

**DOI:** 10.1002/ccr3.9440

**Published:** 2024-09-13

**Authors:** HongJie Chen, Yaodong Han, Wu Li, Jun Zhang, Zhong Liang

**Affiliations:** ^1^ Department of Urology The First Ren Ming Hospital of Lanzhou Lanzhou China; ^2^ Department of Imageology The First Ren Ming Hospital of Lanzhou Lanzhou China

**Keywords:** foreign bodies, missed diagnosis, self‐inserted, urinary bladder

## Abstract

**Key Clinical Message:**

A self‐insertion of the styrofoam in urinary bladder is relatively rare. The diagnosis might be missed due to concealing the history of self‐insertion of the foreign body and the presence of gas in the bladder on CT and MRI. Younger patients with lower urinary tract symptoms should raise the index of suspicion.

**Abstract:**

Transurethral self‐insertion of a foreign body into the bladder is the most common type of bladder foreign body, which is unlikely to be misdiagnosed. we report a case of self‐insertion bladder foreign body and present the symptoms, imaging, diagnosis and treatment in a 14‐year‐old Chinese boy of Han nationality. Younger patients with lower urinary tract symptoms should raise the index of suspicion. Endoscopic removal of foreign bodies can be a challenge. Patients with self‐insertion of foreign objects should undergo psychiatric evaluation to avoid repeated transurethral insertion of foreign bodies.

## 1 INTRODUCTION

In the pediatric population, foreign bodies within the urinary bladder are uncommon. But in the past few decades, there has been an increase in the number of reports of self‐inserted bladder foreign bodies, especially in children and adolescents.^1^ Complications of self‐insertion in the bladder such as urinary retention, dysuria, increased frequency, reduced volume, nocturia, hematuria, painful erections, and pelvic pain are common.^2^ Due to overlapping symptoms with urinary tract infections and knowing the history of patients with this condition can be difficult, these patients are easily misdiagnosed and mistreated.^3^ Here we report a case of self‐inserted bladder foreign bodies in a boy. The diagnosis was missed because of the history of self‐insertion of the foreign body being concealed and the foreign body being difficultly identified on Computed tomography(CT) and Magnetic Resonance Imaging(MRI), which is less reported in the literature.

## 2 CASE HISTORY/EXAMINATION

**FIGURE 1:**
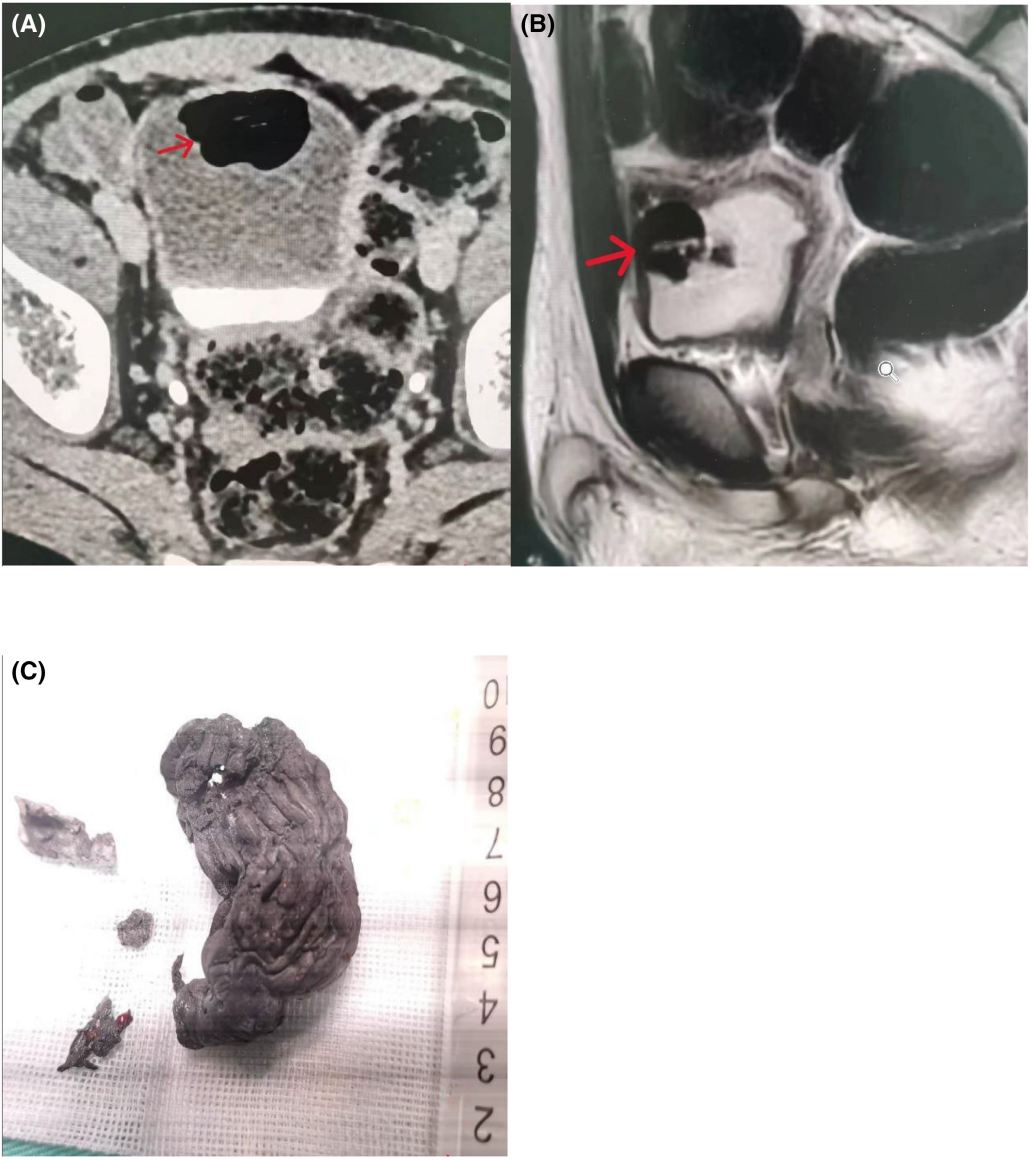
(A) CT showing a lot of air accumulations in the urinary bladder (marked with a red arrow). (B) MRI indicated the air and fluid level in the bladder (red arrow). (C) Retrieved foreign bodies from the urinary bladder.

A 14‐year‐old Chinese boy with hypogastric pain presented with urinary frequency, urgency, incontinence and hematuria for a week. His family history and physical examination did not reveal abnormalities. He had no history of hematuria or psychiatric disorders. Urinalysis showed moderate hematuria (1800 red blood cells/μL), bacteriuria (780 leukocytes/μL). Ultrasonography of abdomen revealed an echogenic shadow on top wall of the bladder. CT scan and MRI showed the bladder wall is manic and thickened, and there was a lot of gas in the bladder (Figure 1A,B).

## 3 METHODS

Our initial diagnosis was bladder contusion and emphysematous cystitis. The patient's clinical symptoms did not improve after 1 week of antibiotic treatment and indwelling urinary catheterization. Further study with a cystoscopy for the patient under general anesthesia revealed two foreign bodies which floating on the top of bladder. Foreign bodies were hard to remove from the urethra, and then the patient underwent open cystotomy with removal of foreign body after obtaining informed consent. After opening the bladder during the surgical procedure, we discovered that two foreign bodies that were grayish white, irregular, and smooth surface.

## 4 CONCLUSION AND RESULTS

The final confirmation of bladder foreign body was spray foam insulation (Figure 1C). On further enquiry after surgery, the child admitted self‐insertion of spray foam insulation 1 month prior urinary incontinence symptoms. The patient was discharged on the third postoperative day. The urethral catheter was removed a week after surgery. The psychologist used DASS‐21 to assess the child's mental health, and the scores of depression, anxiety and stress were moderate. The patient was followed up for 1 month without complications.

## 5 DISCUSSION

Literature is reported in case reports and small case series about self‐inserted bladder foreign bodies, and there is still a lack of data on management and epidemiology in this area. There is no consensus or screening method for such patients presenting to the emergency physicians and urologist. Foreign objects range from common household items such as straws, cotton swabs, hairpins and safety pins to more industrial items such as nails and cables, and so on.^4^ The “styrofoam” is a rare foreign body in the bladder. Our patient watched his father use the substance to fix a leaking fish tank. He sprayed it into his urethra because he had urinary incontinence. Park et al.^5^ reported a case of an adult male with spraying styrofoam into the urethra and bladder because of erectile dysfunction. Bladder foreign bodies can cause hematuria, dysuria, urgency, incontinence and acute urinary retention.

Some behaviors such as autoerotic stimulation, intoxication, senility, psychosis with or without mood disturbance, cognitive disorders, malingering, and stash of drug through the urethra into the bladder to escape prosecution are reported as the cause of the self‐inserted bladder foreign bodies.^6^ Causes in children and adolescents may reflect mental disorders, accidental penetration, sexual stimulation, or simple curiosity.^7^ Our child had mental health problems that were evaluated by a psychologist, which may be as the cause of the self‐inserted bladder foreign bodies. Almost all patients, out of shame and embarrassment, often delay seeking medical assistance until they develop an intolerable symptom such as severe lower urinary tract symptoms, bladder stones, fistulas and even kidney failure.^8^ Our patient concealed the history of self‐insertion of foreign body throughout the diagnosis and treatment, which is an important reason for the misdiagnosis. Another important reason is that substances such as “styrofoam” present as gas in the bladder on CT and MRI. Winot^9^ concluded that there were three reasons for the misdiagnosis of self‐inserted bladder foreign bodies. First, as they are rare, providers may have a low level of suspicion for a foreign body in patients who present with lower urinary tract symptoms. The poor patient history, which may be hesitant to provide due to fear of embarrassment or harassment, was second reason. A third cause of missed diagnosis may result from inconclusive imaging studies. Our patient encountered all the causes of misdiagnosis. For these reason, it is important for those patients to undergo cystoscopy.

The optimal approach to bladder foreign body removal depends on the material, size and form, patient age, the professional skills of the operating surgeon and instruments available.^10^ CT can provide detailed information about foreign bodies and visualize images, and should be an essential means of examination. Reconstructed three‐dimensional(3D)‐CT images can help doctors make better surgical plans.^11^ Most foreign objects in the urinary system can be removed endoscopically and gentle endoscopic management is the primary treatment.^12^ But in the present case, we had finally been selected open surgical removal due to encounter a defeat of cystourethroscopy removed.

Angulo‐Lozano et al.,^13^ found that half of the patients with self‐insertion of foreign objects had a history of mental illness. They thought that it was essential to perform a psychiatric evaluation to diagnose or address any underlying psychiatric conditions that could cause this behavior. If the underlying cause is not recognized, the patient may face repeated injuries. Especially for children and adolescents, to understand the exact cause of foreign body insertion and give correct guidance, as well as the reactions of medical staff during treatment such as confusion, laughter, ridicule, fear, embarrassment and anger, will have an important impact on the psychology of children and adolescents. We believe that in the process of diagnosis and treatment of medical care should be avoided bad emotions in the face of children. In addition, the follow‐up of such patients for psychological counseling is also very necessary.^14^ And contact information should be established in order to understand the patient's mental health.

Self‐insertion of foreign objects are unlikely to be misdiagnosed, and younger patients with lower urinary tract symptoms should raise the index of suspicion. Endoscopic removal of foreign bodies can be a challenge. Patients with self‐insertion of foreign objects should undergo psychiatric evaluation to avoid repeated transurethral insertion of foreign bodies.

## AUTHOR CONTRIBUTIONS

**HongJie Chen:** Resources; writing – original draft. **Yaodong Han:** Writing – original draft. **Wu Li:** Resources. **Jun Zhang:** Investigation. **Zhong Liang:** Resources.

## FUNDING INFORMATION

None.

## CONFLICT OF INTEREST STATEMENT

The authors declare no conflicts of interest.

## CONSENT STATEMENT

Written informed consent was obtained from the patient to publish this report in accordance with the journal's patient consent policy.

## Data Availability

The data that support the findings of this case report are available and can be provided upon request.
